# San Leopoldo Sanitary Establishment, Havana

**Published:** 1848-01

**Authors:** 


### SAN LEOPOLDO SANITARY ESTABLISHMENT, HAVANA.

Under the above title, Dr. Dupierris, of Havana, has opened a house for the reception of invalids. The buildings have a pleasant and healthy situation on the sea shore, near the city; they are of considerable extent, and are surrounded by beautiful walks, and extensive covered galleries. The arrangements for the comfort and pleasure of sojourners there seem to be perfect. Besides this, the director resides in the house, and gives his personal attention to those who require it. Great numbers of our fellow-citizens seek Havana during the winter, and we can heartily recommend to them the San Leopoldo house of health, and its director. Dr. Dupierris is an accomplished and skilful physician, who has resided long at Havana.

